# Supplementary material for: Enhancing Genetic Gains in Grain Yield and Efficiency of Testing Sites of Early-Maturing Maize Hybrids under Contrasting Environments
Source: Genes (Basel). 2023 Sep 30;14(10):1900. doi: 10.3390/genes14101900 (PMC10606723; doi:10.3390/genes14101900)
Supplement: Supplementary file 1 [file genes-14-01900-s001.zip › genes-2594450-supplementary.pdf]

Table S1: List, period, and year of development of the 54 hybrids evaluated across 14 stress and 21 non-stress environments used in the study.

S/N	Variety	Period	Year development
1	TZE-W Pop DT STR x TZEI 19	1	2008
2	(TZEI 63 x TZEI 87) x (TZEI 59 x TZEI 108)	1	2008
3	(TZEI 135 x TZEI 129) x (TZEI 16 x TZEI 157)	1	2008
4	TZE-Y Pop DT STR x TZEI 17	1	2008
5	TZEI 23 x TZEI 13	1	2009
6	(TZEI 63 x TZEI 59) x TZEI 87	1	2008
7	TZE W Pop DT STR C4 x TZEI 22	1	2008
8	TZEI 24 x TZEI 17	1	2008
9	TZEI 16 x TZEI 8	1	2008
10	TZE Y Pop DT STR C4 x TZEI 11	1	2009
11	TZEI 188 x TZEI 98	1	2010
12	TZEI 7 x TZEI 26	1	2009
13	TZEI 31 x TZEI 63	1	2010
14	TZEI 5 x TZEI 98	1	2009
15	TZEI 14 x TZEI 25	1	2009
16	TZEI 31 x TZEI 18	1	2010
17	ENT 12 x TZEI 48	1	2011
18	TZEI 83 x TZEI 60	1	2010
1	TZdEI 352 x TZEI 355	2	2013
2	TZdEI 352 x TZEI 383	2	2013
3	TZEI 352 x TZdEI 352	2	2013
4	TZEI 355 x TZdEI 425	2	2013
5	TZEI 326 x TZdEI 352	2	2013
6	TZE-W Pop DT C5 STR C5 x TZEI 63	2	2013
7	TZEI 326 x TZdEI 425	2	2013
8	TZE-Y Pop DT C5 STR C5 x ENT 13	2	2013
9	TZE-Y Pop DT C5 STR C5 x TZEI 129	2	2013
10	TZEI 495 x ENT 13	2	2013
11	TZEI 470 x ENT 13	2	2012
12	TZEI 474 x TZEI 17	2	2013
13	TZEI 507 x TZEI 129	2	2013
14	TZEI 516 x ENT 13	2	2012
15	TZEI 474 x TZEI 10	2	2012
16	TZEI 486 x TZEI 23	2	2012
17	TZE-Y Pop DT C5 STR C5 x TZEI 17	2	2012
18	TZE-Y Pop DT C5 STR C5 x TZEI 10	2	2012
1	TZdEI 173 x TZdEI 352	3	2014

2	TZdEI 157 x TZdEI 352	3	2014
3	TZdEI 173 x TZdEI 280	3	2014
4	TZdEI 124 x TZdEI 268	3	2014
5	TZdEI 314 x TZdEI 105	3	2014
6	TZdEI 173 x TZdEI 492	3	2015
7	TZdEI 268 x TZdEI 131	3	2014
8	TZdEI 378 x TZdEI 173	3	2015
9	TZdEI 378 x TZdEI 98	3	2015
10	TZdEI 352 x TZdEI 315	3	2015
11	TZEI 18 x TZdEI 352	3	2015
12	TZdEI 479 x TZdEI 124	3	2015
13	TZdEI 352 x TZdEI 441	3	2015
14	TZdEI 17 x TZEI 17	3	2015
15	TZdEI 68 x TZEI 10	3	2015
16	TZdEI 21 x TZEI 23	3	2015
17	TZdEI 24 x TZEI 17	3	2015
18	TZdEI 21 x TZEI 10	3	2015

Supplementary Table S2: Entry number, pedigree, period, environment, and year of evaluation of the top 15, middle 15, and worst 5 hybrids evaluated across 14 stress and 21 non-stress environments used in the study.

Entry	Pedigree	Period	Environment's code	Environment	Year of evaluation
1	(TZEI 63 x TZEI 87) x (TZEI 59 x TZEI 108)	1	E1	Ile-Ife low N	2017
2	(TZEI 135 x TZEI 129) x (TZEI 16 x TZEI 157)	1	E2	Mokwa Striga	2017
3	TZEI 16 x TZEI 8	1	E3	Abuja Striga	2017
4	TZEI 188 x TZEI 98	1	E4	Ina Striga	2017
5	TZEI 14 x TZEI 25	1	E5	Nyankpala Striga	2017
6	TZEI 31 x TZEI 18	1	E6	Ikenne drought	2017
7	ENT 12 x TZEI 48	1	E7	Ikenne drought	2018
8	TZdEI 173 x TZdEI 352	2	E8	Ile-Ife low N	2018
9	TZdEI 157 x TZdEI 352	2	E9	Abuja Striga	2018
10	TZdEI 173 x TZdEI 280	2	E10	Mokwa Striga	2018
11	TZdEI 124 x TZdEI 268	2	E11	Mokwa low N	2018
12	TZdEI 314 x TZdEI 105	2	E12	Ile-Ife low N	2019
13	TZdEI 173 x TZdEI 492	2	E13	Mokwa Striga	2019
14	TZdEI 268 x TZdEI 131	2	E14	Ikenne drought	2019
15	TZdEI 378 x TZdEI 173	2	E15	Ikenne optimal	2017
16	TZdEI 378 x TZdEI 98	2	E16	Ile-Ife high N	2017
17	TZdEI 352 x TZdEI 315	2	E17	Mokwa high N	2017
18	TZEI 18 x TZdEI 352	2	E18	Abuja optimal	2017
19	TZdEI 479 x TZdEI 124	2	E19	Bagauda optimal	2017
20	TZdEI 17 x TZEI 17	2	E20	Zaria optimal	2017
21	TZdEI 68 x TZEI 10	2	E21	Angaradebou optimal	2017
22	TZdEI 21 x TZEI 23	2	E22	Manga optimal	2017
23	TZdEI 24 x TZEI 17	2	E23	Nyankpala optimal	2017
24	TZdEI 21 x TZEI 10	2	E24	Ejura optimal	2017
25	TZdEI 352 x TZEI 355	3	E25	Ina optimal	2017
26	TZdEI 352 x TZEI 383	3	E26	Ina Striga	2017
27	TZEI 352 x TZdEI 352	3	E27	Ikenne optimal	2018
28	TZEI 326 x TZdEI 352	3	E28	Bagauda optimal	2018
29	TZE-W Pop DT C5 STR C5 x TZEI 63	3	E29	Abuja optimal	2018
30	TZE-Y Pop DT C5 STR C5 x ENT 13	3	E30	Ele-Ife high N	2018
31	TZE-Y Pop DT C5 STR C5 x TZEI 129	3	E31	Mokwa high N	2018
32	TZEI 495 x ENT 13	3	E32	Zaria optimal	2018
33	TZEI 474 x TZEI 17	3	E33	Ife high N 19	2019
34	TZEI 486 x TZEI 23	3	E34	Abuja optimal	2019
35	TZE-Y Pop DT C5 STR C5 x TZEI 17	3	E35	Mokwa optimal	2019
